# Transient Receptor Potential Vanilloid 4 Ion Channel Functions as a Pruriceptor in Epidermal Keratinocytes to Evoke Histaminergic Itch*

**DOI:** 10.1074/jbc.M116.716464

**Published:** 2016-03-09

**Authors:** Yong Chen, Quan Fang, Zilong Wang, Jennifer Y. Zhang, Amanda S. MacLeod, Russell P. Hall, Wolfgang B. Liedtke

**Affiliations:** From the Departments of ‡ Neurology,; § Dermatology,; ¶ Neurobiology, and; ‖ Anesthesiology, Duke University Medical Center, Durham, North Carolina 27710 and; ** Neurology Clinics for Headache, Head Pain and Trigeminal Sensory Disorders, Duke University Medical Center, Durham, North Carolina 27705

**Keywords:** calcium, extracellular-signal-regulated kinase (ERK), inhibitor, keratinocyte, skin, TRPV4, histaminergic itch, pruritogen

## Abstract

TRPV4 ion channels function in epidermal keratinocytes and in innervating sensory neurons; however, the contribution of the channel in either cell to neurosensory function remains to be elucidated. We recently reported TRPV4 as a critical component of the keratinocyte machinery that responds to ultraviolet B (UVB) and functions critically to convert the keratinocyte into a pain-generator cell after excess UVB exposure. One key mechanism in keratinocytes was increased expression and secretion of endothelin-1, which is also a known pruritogen. Here we address the question of whether TRPV4 in skin keratinocytes functions in itch, as a particular form of “forefront” signaling in non-neural cells. Our results support this novel concept based on attenuated scratching behavior in response to histaminergic (histamine, compound 48/80, endothelin-1), not non-histaminergic (chloroquine) pruritogens in *Trpv4* keratinocyte-specific and inducible knock-out mice. We demonstrate that keratinocytes rely on TRPV4 for calcium influx in response to histaminergic pruritogens. TRPV4 activation in keratinocytes evokes phosphorylation of mitogen-activated protein kinase, ERK, for histaminergic pruritogens. This finding is relevant because we observed robust anti-pruritic effects with topical applications of selective inhibitors for TRPV4 and also for MEK, the kinase upstream of ERK, suggesting that calcium influx via TRPV4 in keratinocytes leads to ERK-phosphorylation, which in turn rapidly converts the keratinocyte into an organismal itch-generator cell. In support of this concept we found that scratching behavior, evoked by direct intradermal activation of TRPV4, was critically dependent on TRPV4 expression in keratinocytes. Thus, TRPV4 functions as a pruriceptor-TRP in skin keratinocytes in histaminergic itch, a novel basic concept with translational-medical relevance.

## Introduction

Itch is a clinical problem that leaves many sufferers insufficiently treated, with >20 million in the United States (1–3). This is also caused by incomplete understanding of its molecular, cellular, and cell-to-cell signaling mechanisms. Neural pathways have been understood as key for itch, whereby specialized primary sensory pruriceptor neurons relay sensory afferent information to itch-transmitting neural pathways, ultimately evoking the sensation of itch (2, 4–8). Exogenous or endogenous pruritogens are thought to act on primary sensory neurons, producing the sensation of itch by activating the pruriceptors expressed by these afferents. Primary pruriceptor neurons may receive modulatory signals from atopic inflammatory cells, such as mast cells, and also from epidermal keratinocytes (7). It was recently elucidated that the atopia cytokine, thymic stromal lymphopoietin (TSLP), was secreted from skin keratinocytes to activate TRPA1 ion channels on primary pruriceptor neurons and induced itch (9). Despite this landmark discovery, mechanisms of how the epidermal keratinocyte specifically functions to evoke itch remain largely unknown, especially mechanistic insights that rely on precise genetic targeting of genes-of-interest only in keratinocytes. In other words, molecular and cell-to-cell signaling mechanisms of forefront pruri-transduction are elusive.

We recently defined a mechanism of how ultraviolet B (UVB)^3^ radiation activates TRPV4 ion channels in skin epidermal keratinocytes (10). Their genetically encoded, inducible absence in skin keratinocytes suffices to contain pain and tissue damage evoked by UVB overexposure. In skin keratinocytes, TRPV4 activation by UVB is potentiated by endothelin-1 (ET-1) via endothelin receptors A and B. TRPV4-activation in these cells leads to Ca^2+^ influx, which in turn increases gene expression of ET-1, providing the substrate of a feed-forward mechanism that sustains organismal pain. This is an interesting observation in the context of itch because ET-1 injection into skin is known to cause itch in human subjects and evokes scratching behavior in experimental animals upon intradermal injection (11–16). TRPV4 has been implicated in other forms of pain (10, 17–26). It is a multimodally activated TRPV channel, *e.g.* activated by changes in osmotic pressure, mechanical, UVB, and chemical cues and modified by thermal cues (27–31). Except for the recent elucidation of the role of TRPV4 as ionotropic receptor for UVB in keratinocytes to reprogram these cells into organismal pain generators, its role in pain has been attributed to its expression in primary sensory neurons.

Against this background, especially the finding of TRPV4-dependent secretion of the pruritogen, ET-1, by keratinocytes, we felt that we have raised a timely question, namely whether TRPV4 plays a role in itch, in particular whether TRPV4 in keratinocytes of the epidermis can drive scratching behavior. To address this question we decided to first focus on acute itch and, specifically, as an initial priority, to examine prototypic examples of histaminergic itch, including ET-1-evoked itch, plus chloroquine-caused non-histaminergic itch. In this study we are reporting an exciting new function of TRPV4 in forefront signaling of the integument, namely that TRPV4 in epidermal keratinocytes functions as a pruriceptor-TRP channel in acute histaminergic itch, including itch evoked by ET-1, not in non-histaminergic itch evoked by chloroquine. Direct activation of TRPV4 channels also evokes scratching behavior, which appears completely dependent on TRPV4 expression in keratinocytes, thus underscoring the role of this cell and its expression of TRPV4 in itch. Complementing findings in our *Trpv4* keratinocyte-specific inducible knock-out (*Trpv4* cKO) mice, we demonstrate Ca^2+^ transients in response to histaminergic pruritogens in cultured primary keratinocytes that depend on TRPV4. Ca^2+^ influx via TRPV4 then up-regulates phosphorylation of the mitogen-activated protein kinase ERK in keratinocytes. Consequently, we find topical transdermal treatment with a selective inhibitor of TRPV4 to function efficiently as an anti-pruritogen. Moreover, we observed similar *in vivo* anti-pruritic effects when topically targeting MEK, upstream of ERK, with a selective inhibitor.

## Experimental Procedures

### 

#### 

##### Animals

The pan-null phenotype of *Trpv4*^−/−^ mice relies on excision of the exon encoding transmembrane domains 5–6. Mice were outcrossed to C57BL/6J background and PCR-genotyped (10, 25, 26, 32). Male WT (C57BL/6J) and *Trpv4*^−/−^, 2–2.5 months of age, were used for all experiments.

**FIGURE 1.**
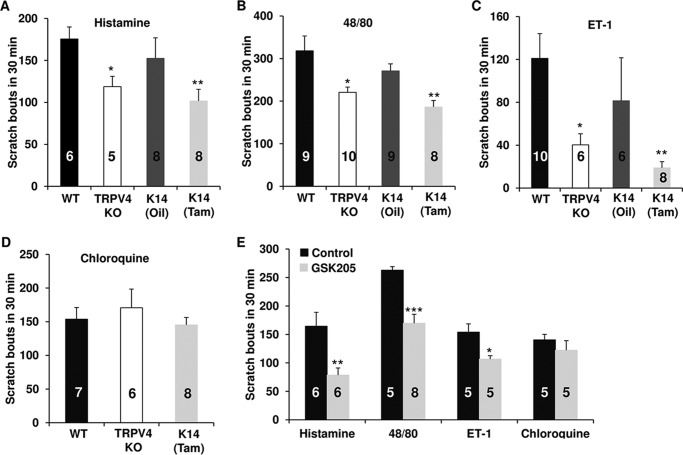
***Trpv4* in skin keratinocytes is essential for histamine-dependent itch.** Histamine (*A*), compound 48/80 (*B*), and ET-1 (*C*), but not chloroquine (*D*), evoked acute scratching behaviors that were significantly attenuated in *Trpv4* cKO (K14-Tam) and pan-null mice (TRPV4 KO) *versus* their respective controls (*A–D*, *, *p* < 0.05; **, *p* < 0.01 *versus* WT). Mice topically transdermally treated with the TRPV4-selective inhibitor GSK205 showed a significant reduction of scratching behaviors (*E*, *, *p* < 0.05; **, *p* < 0.01; ***, *p* < 0.001 *versus Control*). One-way analysis of variance with Tukey's post hoc test was used for *A–D*, and two-tail *t* test was used for *E*. Group size is indicated in the *bars*.

Keratinocyte-specific, tamoxifen (tam)-inducible *Trpv4* knockdown mice were used as previously described (10). In brief, the *Trpv4* genomic locus was engineered so that loxP sites surrounded exon 13, which encodes TM5–6. This mutation was propagated in mice that were crossed to K14-CRE-ER^tam^ mice, so that *Trpv4*^lox/lox^x(K14-CRE-ER^tam^) mice could be induced by tam (Sigma) administration via oral gavage for five consecutive days at 6 mg/day in 0.3 ml corn oil at age 2–2.5 months of age, plus a 1-time booster 2 weeks after the last application. Control animals received the same volume of corn oil. Efficiency of targeting was verified by quantitative real-time PCR and immunohistochemistry for *Trpv4* expression in skin at gene and protein levels, respectively (10). Both male and female mice were used for *in vivo* scratching behavior as shown in Figs. 1 and 5, and no difference was detected between sexes.

Animals were housed in climate-controlled rooms on a 12/12-h light/dark cycle with water and a standardized rodent diet available *ad libitum*. All animal protocols were approved by the Duke University Institutional Animal Care and Use Committee in compliance with National Institutes of Health guidelines.

##### Drugs

Histamine, compound 48/80, endothelin-1, chloroquine, and GSK1016790A (GSK101) were purchased from Sigma. GSK2193874 (GSK219) was obtained from Tocris, U0126 was from Selleckchem, and GSK205 was synthesized (26, 33). All were dissolved in sterile normal saline except that GSK101 and GSK205 were dissolved in DMSO (20 mm in stock) and further diluted until use.

##### Topical Treatment Formulation

Compounds GSK205 and U0126 were kept as DMSO stock, then diluted to 100 μm and 0.1 mg/ml, respectively, in 25% isopropyl alcohol, 15% ethanol, and 60% glycerol when used.

##### Itch Behavioral Tests

Mice were shaved at the dorsal neck where intradermal injections and topical applications were applied. Mice were allowed to acclimate to a Plexiglas chamber for at least 30 min before testing and received intradermal injection of pruritogens (histamine, 500 μg/50 μl; 48/80, 100 μg/50 μl; ET-1, 25 ng/50 μl; chloroquine, 200 μg/50 μl) or saline through a 30-gauge needle into the nape of neck to elicit scratching behavior. After injection, mice were immediately placed back in the chamber, and the scratching behavior was recorded by a Panasonic video camera for a 30-min observation period. Hind limb scratching behavior directed toward the shaved area at the nape of neck was observed. One scratch is defined as a lifting of the hind limb toward the injection site and then a replacing of the limb back to the floor, regardless of how many scratching strokes take place between those two movements. Behavioral analysis was conducted by observers blinded to genotype.

To investigate the topical effects of the specific TRPV4 inhibitor GSK205 or the specific MEK inhibitor U0126 on pruritogen-induced scratching behaviors, mice received a transdermal-topical application of 100 μl of formulated GSK205 (100 μm) or U0126 (0.1 mg/ml) on the shaved area at the nape of neck 20 min before pruritogen injections. Control animals received the same volume of placebo.

##### Keratinocytes Culture and Ca^2+^ Imaging

Primary mouse keratinocytes were cultured following previous protocol (10). The epidermis from the back skin of newborn WT mice (P0-P2) was separated from the dermis by floating the skin on 0.25% trypsin (Gibco) for 14–18 h at 4 °C. Basal keratinocytes were separated from the cornified sheets by filtration through a 70 μm cell strainer (BD Biosciences). Keratinocytes were plated on collagen-coated dishes or glass coverslips and grown in EME media (Gibco) supplemented with bovine pituitary extract and epidermal growth factor, 10% chelexed fetal bovine serum (Gibco), 100 pmol of cholera toxin (Calbiochem), and 1× antibiotics/antimycotics (Gibco) in an incubator at 5% CO_2_ and 37 °C.

Primary human keratinocytes were cultured as previously described (34). In brief, surgically discarded foreskin samples, obtained from Duke Children's Hospital in accordance to institutionally approved IRB protocol, were incubated with Dispase (Gibco, 4 units/ml) for 12–16 h at 4 °C followed by 0.05% trypsin (Gibco) for 10–20 min at 37 °C. Cells were grown and passaged in keratinocyte serum-free media (Invitrogen) at 37 °C with 5% CO_2_ and used at passage 2–3.

Ca^2+^ imaging of primary epidermal keratinocytes in response to chemicals was conducted after loading with 2 μm fura2-AM (Invitrogen) for 30 min after a ratiometric Ca^2+^-imaging protocol with 340/380-nm blue light for dual excitation. Ratios of emissions were acquired at 0.5 Hz. Δ*R*/*R*_0_ was determined as the fraction of the increase of a given ratio over baseline ratio divided by baseline ratio.

To investigate the effects of the specific TRPV4 inhibitors GSK205 or GSK219 on pruritogen-induced Ca^2+^ influx and pERK expression, cells were incubated with GSK205 or GSK219 for 15 min before stimulation. Control cells received the same volume of vehicle.

##### Western Blot

Routine procedures were followed (10, 25, 26, 35). Briefly, cultured keratinocytes and dissected dorsal skin (0.5 × 0.5 cm, the area that received the treatment) were protein-extracted in radioimmunoprecipitation assay (RIPA, Sigma) buffer and electroblotted to nitrocellulose membranes after gel separation of proteins in a 4–15% polyacrylamide gel (Bio-Rad). Membranes were blocked with 5% BSA (Sigma) in TBST, and pERK and ERK were specifically detected with primary antibodies (rabbit anti-pERK (catalog #9101) and anti-ERK (catalog #4695), both at 1:2000; Cell Signaling Technology), secondary antibody (anti-rabbit peroxidase-conjugated, 1:5000; Jackson ImmunoResearch), and chemiluminescence substrate (ECL-Advance, GE Healthcare). Abundance was quantified using ImagePro Plus software. β-Actin, as a control, was detected with a mouse monoclonal anti-β-actin antibody (1:4000; catalog #sc-47778, Santa Cruz) or a rabbit polyclonal anti-β-actin antibody (1:4000; catalog #A5316, Sigma). Immunoblot band intensity was quantitated using the software Image J (National Institutes of Health).

##### Statistical Analysis

All data are expressed as the mean ± S.E. Two-tailed *t* tests or one-way analysis of variance followed by Tukey's post hoc test were used for group comparisons. *p* < 0.05 indicated statistically significant differences.

## Results

### 

#### 

##### Trpv4 in Skin Keratinocyte Is Critical for Histaminergic Itch

To assess the contribution of keratinocyte TRPV4 channels to acute itch, we subjected *Trpv4* cKO mice to intradermal injections of both histaminergic and non-histaminergic pruritogens. Throughout, we also challenged *Trpv4* pan-null mice in order to be able to compare any eventual behavioral phenotype present in *Trpv4* cKO mice with that in the respective pan-null mouse. All histaminergic pruritogens including ET-1 evoked a solid scratching response, namely histamine itself (Fig. 1*A*), the polymeric secretagogue and MrgprX2 activator, compound 48/80 (36–40) (Fig. 1*B*), and the partial histaminergic ET-1 (13, 16, 40–43) (Fig. 1*C*), as did the non-histaminergic chloroquine (40, 44–46) (Fig. 1*D*). The scratching responses evoked by histaminergic pruritogens were significantly attenuated in *Trpv4* cKO mice, most robustly for ET-1. In contrast, scratching in response to chloroquine was not (Fig. 1, *A–D*). This means that TRPV4 ion channels in skin keratinocytes powerfully control organismal itch-related scratching behavior by converting the epidermal keratinocyte into an itch-generator cell that directly or indirectly signals to peripheral pruriceptor sensory neurons. In keeping with this new basic concept, *Trpv4* pan-null mice had a similar profile (Fig. 1, *A–D*), their reduced scratching in response to histaminergic pruritogens, indicating that genetically encoded pan-organismal absence of *Trpv4* renders these mice less sensitive to histaminergic pruritogens. We alsotopically applied TRPV4-specific small-molecule inhibitor, GSK205 (10, 33), to mouse epidermis. As a result, histaminergic, but not non-histaminergic, scratching was significantly attenuated (Fig. 1*E*), reiterating the conclusion derived from *Trpv4* cKO mice, namely that TRPV4 channels in epidermal keratinocytes are significant molecular actuators of organismal itch, driving the keratinocyte as itch generator cells. In addition, the experiments with topically applied TRPV4 blocking compound point toward a role for TRPV4 ion channel function as a critical contributor, not only reduced expression of the TRPV4 protein.

##### TRPV4 Is Required for Histaminergic-dependent Pruritogens-induced Ca^2+^ Influx in Keratinocytes

**FIGURE 2.**
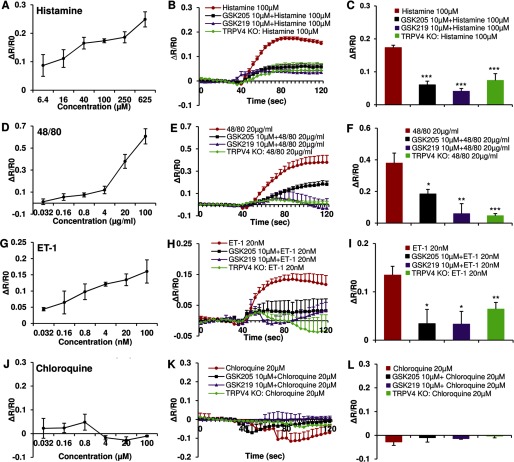
**Histamine-dependent pruritogens evoke Ca^2+^ influx in cultured keratinocytes via TRPV4 channels.** Histamine-dependent pruritogens evoke Ca^2+^ influx in cultured keratinocytes via TRPV4 channels. Histamine (*A*), compound 48/80 (*D*), and the partial histaminergic ET-1 (*G*), but not chloroquine (*J*), triggered Ca^2+^ influx in a dose-dependent manner in keratinocytes. The evoked Ca2^2+^ signal was attenuated in cells pretreated with GSK205 or GSK219, both TRPV4-selective inhibitors, and also in cells from *Trpv4*^−/−^ mice (*B* and *C*, *E* and *F*, *H* and *I*, and *K* and *L*; *, *p* < 0.05; **, *p* < 0.01; ***, *p* < 0.001 *versus* the respective pruritogens). Two-tail *t* test was used for statistic analyses. *n* = 150–300 cells/treatment.

**FIGURE 3.**
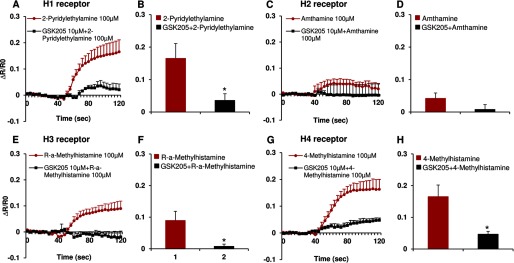
**Histamine receptor agonists induce Ca^2+^ influx in cultured murine keratinocytes via TRPV4 channels.** 2-Pyridylethylamine (selective H1 receptor agonist, *A* and *B*), and 4-methylhistamine (selective H4 receptor agonist, *G* and *H*), but not amthamine (selective H2 receptor agonist, *C* and *D*), evoked Ca^2+^ influx in murine keratinocytes, which was attenuated by pretreatment with TRPV4 inhibitor, GSK205. For selective activation of H3 receptor with *R*-α-methylhistamine (selective H3 receptor agonist, *E* and *F*), the increase were less robust. Two-tail *t* test was used for statistic analyses (*B*, *D*, *F*, and *H*; *, *p* < 0.05 *versus* agonists). *n* = 100–200 cells/treatment.

**FIGURE 4.**
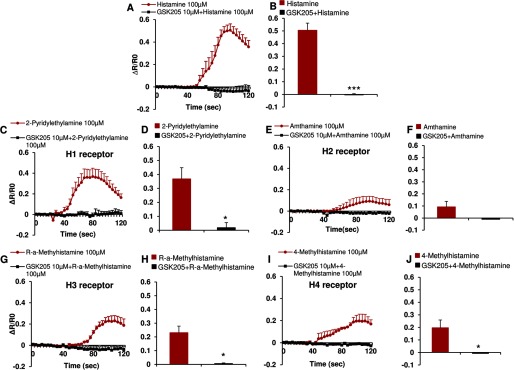
**Histamine receptor agonists induce Ca^2+^ influx in cultured human keratinocytes via TRPV4 channels.** Histamine (non-selective H receptor agonist, *A* and *B*), 2-pyridylethylamine (selective H1 receptor agonist, *C* and *D*), *R*-α-methylhistamine (selective H3 receptor agonist, *G* and *H*), and 4-methylhistamine (selective H4 receptor agonist, *I* and *J*), but not amthamine (selective H2 receptor agonist, *E* and *F*), evoked Ca^2+^ influx in a dose-dependent manner in human keratinocytes, which was attenuated by pretreatment with TRPV4 inhibitor, GSK205. A two-tail *t* test was used for statistic analyses (*A*, *D*, *F*, *H*, and *J*; ***, *p* < 0.0001; *, *p* < 0.05 *versus* agonists). *n* = 100–200 cells/treatment.

These *in vivo* results raise the question of how the histaminergic pruritogens that we have used to evoke TRPV4-dependent scratching affect Ca^2+^ signaling in epidermal keratinocyes given that TRPV4 is known to function as a Ca^2+^-permeable TRP channel in these cells (47). To address this specific question, we stimulated primary murine keratinocytes with the same pruritogens used *in vivo*, then asked whether inhibiting TRPV4 channel activity with a selective small-molecule inhibitor would attenuate any resulting Ca^2+^ transients. We first used the classic pruritogen, histamine, which resulted in a dose-dependent Ca^2+^ signal, strongly attenuated by two selective TRPV4 inhibitors, GSK205 and GSK219 (Fig. 2, *A–C*). We obtained a similar reduction of Ca^2+^ signal when stimulating cultured keratinocytes derived from *Trpv4*^−/−^ pan-null mice, confirming the critical role of TRPV4 in Ca^2+^ influx downstream of histamine-receptor signaling. Given the significance of histamine for itch, we also elucidated the receptor subtype present in keratinocytes. Our results suggest histamine receptors of the H1, H3, and H4 subtype to be appreciably involved, not H2 receptors. This finding is in keeping with previously established expression patterns in keratinocytes (48–51) and illustrated in Fig. 3. These three histamine receptor subtypes signaled to TRPV4 as their selective stimulation led to an appreciable Ca^2+^ transient in primary keratinocytes that could be virtually eliminated by GSK205, as also illustrated in Fig. 3. Given the translational medical relevance of this finding, we recapitulated this experiment in primary human keratinocytes. Results are shown in Fig. 4, demonstrating a similar capability of histamine to evoke Ca^2+^ transients in primary human keratinocytes. These Ca^2+^ transients could be completely eliminated with GSK205, as shown in Fig. 4. Our findings indicate the TRPV4-mediated Ca^2+^ signal to rely on H1, H3, and H4 receptors. Congruency of mouse human histamine-TRPV4 signaling suggests an evolutionary conserved allergic inflammation mechanism that underlies integumental signaling from keratinocytes to sensory neurons.

Regarding Ca^2+^ signaling in response to other histaminergic pruritogens, we found that compound 48/80 (Fig. 2, *D–F*) and also the partial histaminergic ET-1 (Fig. 2, *G–I*) evoked Ca^2+^ transients in mouse primary keratinocytes in a dose-dependent manner that could be blocked with GSK205 and GSK219 or were dramatically reduced in keratinocytes derived from *Trpv4*^−/−^ pan-null mice. Chloroquine, a non-histaminergic pruritogen, however, did not evoke a Ca^2+^ signal in keratinocytes (Fig. 2, *J–L*).

Taken together, we detected a TRPV4-dependent Ca^2+^ signal in cultured epidermal keratinocytes in response to histaminergic pruritogens. In view of our *in vivo* findings with *Trpv4* cKO mice, we reason that this Ca^2+^ signal is the cellular signaling correlate of histaminergic pruritogen-activation of TRPV4 channels in epidermal keratinocytes, which co-contributes significantly to scratching behavior *in vivo*.

##### GSK101, a TRPV4-selective Agonist, Elicits Scratching-behavior Dependent on TRPV4 Expression in Keratinocytes

**FIGURE 5.**
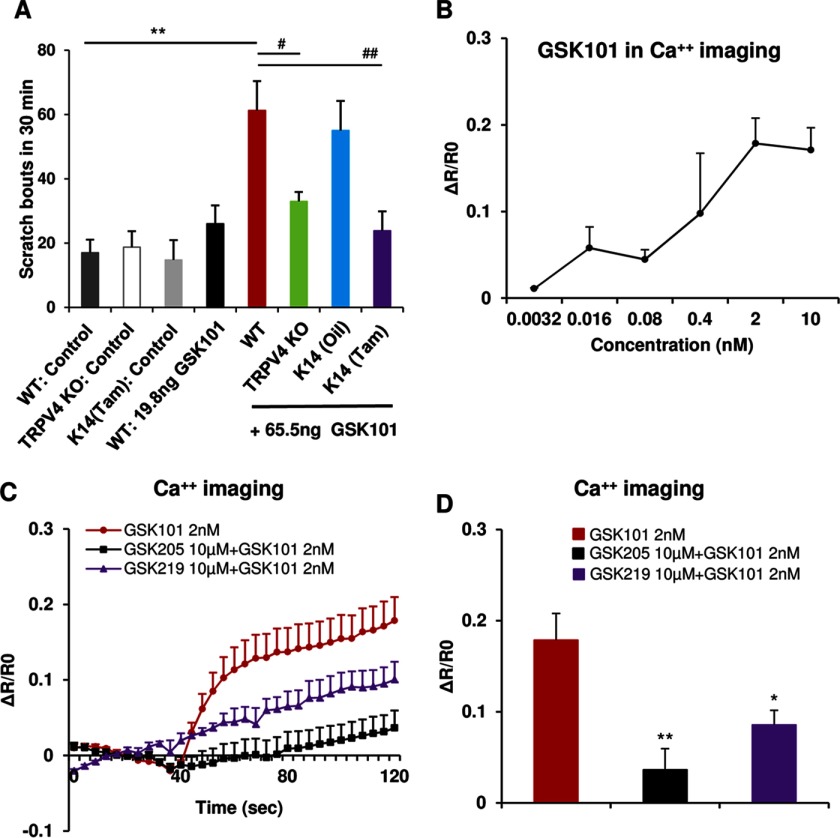
**GSK101 elicits scratching behaviors and triggers Ca^2+^ influx in cultured keratinocytes.** Animals injected with the TRPV4-selective activator, GSK101, displayed a significant scratching response that was attenuated in *Trpv4* pan-null mice. Importantly, scratching behavior depended on TRPV4 expression in keratinocytes, evidenced by a complete lack of response to GSK101 in *Trpv4* cKO mice (*A*, **, *p* < 0.01; #, *p* < 0.05; ##, *p* < 0.01). GSK101 evoked a Ca^2+^ response in a dose-dependent manner in keratinocytes (*B*). *C* and *D* illustrate the keratinocyte Ca^2+^ signal evoked by 2 nm GSK101 and its attenuation by TRPV4-selective inhibitors, GSK205 or GSK219 (*, *p* < 0.05; **, *p* < 0.01 *versus* GSK101). One-way analysis of variance with Tukey's post hoc test was used for *A*, and a two-tail *t* test was used for *D. n* = 4–5 mice/group (*A*) and *n* = 150–300 cells/treatment (*B–D*).

Having recorded results that suggest (i) the *Trpv4* gene is necessary for scratching behavior in response to histaminergic pruritogens, (ii) TRPV4 channels in epidermal keratinocytes are necessary for these behaviors, and (iii) these channels in epidermal keratinocytes are activated by the G protein-coupled receptors for the respective pruritogens, we next asked the question of sufficiency of TRPV4 activation for scratching behavior. We documented that increasing concentrations of small-molecule-selective TRPV4 activator, GSK101, evoked itch behavior with increasing frequency (Fig. 5*A*). In a *Trpv4* pan-null knock-out mouse, there was only a marginal, non-significant increase in scratching behavior in response to 65 ng of GSK101 *versus* WT or *Trpv4* pan-null knock-out control, indicating minimal off-target effects of GSK101 as a pruritogen *in vivo*. Thus, scratching behavior, at the organismal level, can be evoked by selective activation of TRPV4. Importantly, we verified that oil-induced *Trpv4* cKO mice scratched not differently from WT mice in response to GSK101. We also showed that *Trpv4* cKO mice induced with tamoxifen showed no significant increase in scratching behavior in response to intradermal injection of GSK101 *versus* WT or *Trpv4* cKO control (Fig. 5*A*). This finding is highly relevant because it suggests that direct activation of TRPV4 channels, expressed by keratinocytes, by intradermal injection of GSK101 leads to scratching behavior in live animals. This is critically dependent on TRPV4 expression by skin keratinocytes. Therefore, we decided next to assess keratinocyte response to selective activation of TRPV4. We did record a dose-response relationship of the resulting Ca^2+^ signal to GSK101 concentrations (Fig. 5*B*). This signal could be significantly attenuated when using two TRPV4-selective inhibitors, GSK205 or GSK219 (Fig. 5, *C* and *D*). Overall, these GSK101-related findings leave open the possibility of a co-contributory role of TRPV4 signaling in sensory neurons or other itch-relevant cells but together with our data, as presented in Figs. 1–4, make the case for an important role for TRPV4 in epidermal forefront signaling as a “pruriceptor-TRP” ion channel in epidermal keratinocytes.

##### ERK Signaling Downstream of TRPV4 in Skin Keratinocyte Is Essential for Histaminergic Itch

**FIGURE 6.**
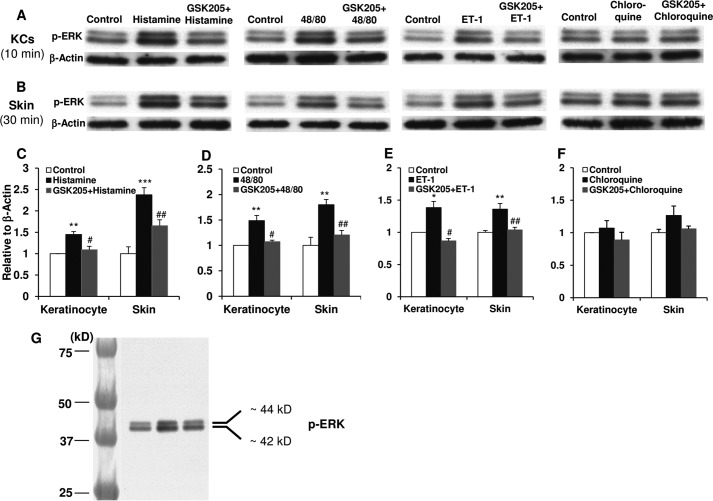
***Trpv4* is essential for the increase of phospho-ERK (*pERK*) in skin keratinocytes (*KCs*) and the integument from live animals in response to histamine-dependent pruritogens.** Western blotting shows pERK expression in cultured keratinocytes (*A*) and nape skin (*B*). *Bar graphs* depict quantitation, demonstrating a significant increase of pERK evoked by histamine (*C*), compound 48/80 (*D*), and ET-1 (*E*), but not chloroquine (*F*) (*, *p* < 0.05; **, *p* < 0.01; ***, *p* < 0.001: pruritogen *versus Control*). Importantly, a significant increase of pERK depends on TRPV4, demonstrated by its reduction to control levels by pretreatment with TRPV4-selective inhibitor GSK205 (*C–F*: #, *p* < 0.05; ##, *p* < 0.01: GSK205+pruritogen *versus* pruritogen). *G* shows Western blots of pERK with a standard molecular mass marker. Two-tail *t* test was used for statistic analyses. *n* = 3 cultures/group for the cultured keratinocytes and *n* = 5∼6 mice/group for skin.

**FIGURE 7.**
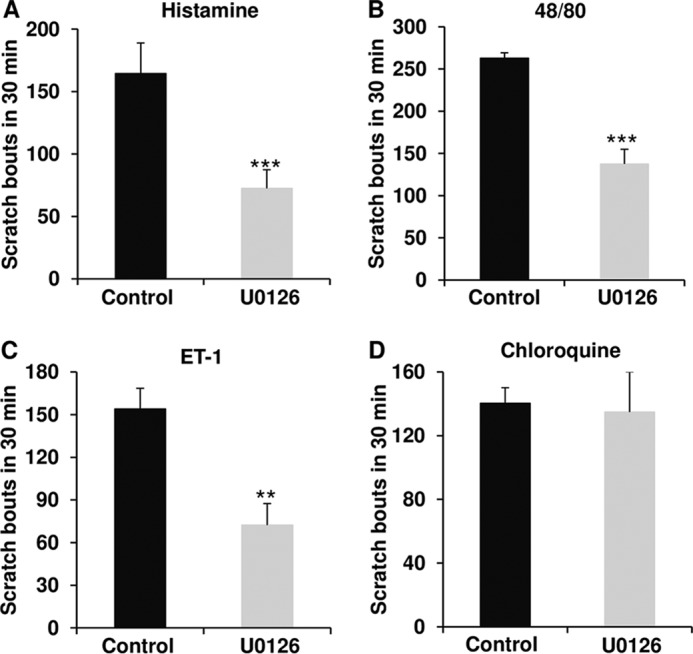
**ERK signaling downstream of TRPV4 is relevant for histaminergic pruritogen-evoked scratching behaviors.** Histamine (*A*), compound 48/80 (*B*), ET-1 (*C*), but not chloroquine (*D*) cause scratching behaviors that are significantly attenuated in mice topically pretreated with the MEK selective inhibitor U0126 (**, *p* < 0.01; ***, *p* < 0.001 *versus Control*). Two-tail *t* test was used for statistic analyses. *n* = 5–6 mice/group.

We next addressed the question of what signals intracellularly in epidermal keratinocytes, downstream of TRPV4-mediated Ca^2+^ influx. Choosing a candidate approach, we focused on mitogen-activated protein kinase signaling of the MEK-ERK pathway based on previous results in an epithelial cell type, upper airway respiratory epithelia, that also provides organismal barrier protection in which we demonstrated MEK-ERK activation in response to an environmental irritant (35, 52). We first probed whether there is rapid ERK phosphorylation in response to histaminergic pruritogens. We recorded affirmative results at the 10-min time point in cultured keratinocytes and the 30-min time point in skin (dermis-epidermis) for all three histaminergic pruritogens tested, not for non-histaminergic pruritogen chloroquine (Fig. 6). The level of total ERK in both cultured cells and skin was not altered (data not shown). We then next addressed whether this rapid increase in ERK phosphorylation depends on TRPV4 by applying the TRPV4-inhibitor, GSK205. In primary keratinocytes, we observed a complete reversal to non-stimulated levels of pERK. In skin from mice challenged *in vivo*, we detected a similar response (Fig. 6). These findings suggest that pERK activation is down-stream of TRPV4-mediated Ca^2+^ influx. To determine the relevance of MEK-ERK phosphorylation for scratching behavior, we applied a selective inhibitor of MEK, U0126, in a topical formulation to skin. In response to histaminergic pruritogens we observed a significant anti-pruritic effect of topical U0126 when mice were intradermally treated and a lack thereof for non-histaminergic pruritogen, chloroquine (Fig. 7).

## Discussion

In this study we describe a novel role for TRPV4 channels in histaminergic itch including ET-1-evoked itch. Importantly, TRPV4 expression and function in epidermal keratinocytes shows a robust contributory role to scratching behavior evoked by histaminergic pruritogens, not for the non-histaminergic chloroquine. This means that keratinocytes of the integument can function as itch generator cells and that TRPV4 plays a significant signaling role in these cells in mediating histaminergic itch. Importantly, direct activation of TRPV4 by intra-dermal injection of TRPV4 activator, GSK101, led to scratching behavior, which critically depended on TRPV4 expression in keratinocytes. This finding underscores the fundamental, hitherto unrecognized role of TRPV4 channels in epidermal keratinocytes in acute histaminergic itch. We recorded complementary findings in primary keratinocyte culture where we observed Ca^2+^ transients in response to the same diverse histaminergic pruritogens that elicit scratching behavior dependent on keratinocyte-TRPV4. We found this Ca^2+^ response to be mediated by TRPV4, which was activated by the respective pruritogens and their cognate keratinocyte-G protein-coupled receptors or directly via selective chemical activator. In epidermal keratinocytes, Ca^2+^ influx via TRPV4 elicits ERK phosphorylation as a downstream signaling event of the forefront pruritogen signaling discovered here. Topical transdermal inhibition of TRPV4 and its downstream kinase target, MEK, which functions upstream of ERK phosphorylation, both showed robust anti-pruritic efficiency in mice challenged with histaminergic pruritogens. Activation of MEK-ERK signaling in keratinocytes is also known from non-itching skin conditions (53, 54). We speculate that MEK-ERK activation by TRPV4 could be the important explanatory difference. This hypothetic concept will have to be tested in future studies. Our results argue for a novel translational medical path of topical treatments to skin that target molecularly defined signaling mechanisms that modulate sensory transduction (55).

In this paper we focus first on acute itch and the role of histaminergic pruritogens. Additional pruritogens need to be studied in the future, with particular focus on chronic itch, a medically relevant condition because of its prevalence and substantial unmet medical need. A central question that we have not addressed in this study is, What specific cellular and molecular mechanisms of cell-to-cell communication do epidermal keratinocytes employ? How does the histaminergic pruritogen-G protein-coupled receptor-TRPV4-Ca^2+^-pERK pathway evoke these signaling mechanisms, and how does this trigger pruriceptor sensory neurons to transmit the signal toward the nervous system? We hypothesize that soluble factors play such roles, possibly proteins, peptides, small-molecule phospholipids, and lipid molecules that are released from keratinocytes to affect innervating peripheral nerve endings of pruriceptor neurons perhaps either via direct keratinocyte nerve fiber signaling or rather indirectly via involvement of immune, vascular, and other adjacent cells.

A recent study examined the response of spinal cord dorsal horn relay neurons to intradermal injection of ET-1 (16). The study reports that ET-1-sensitive neurons respond to multiple modalities yet that >50% respond to spinal superperfusion of the peptide bombesin, which can activate spinal gastrin-releasing peptide receptors known to function in itch circuits, thus identifying these neurons as part of a specific itch circuit that relies on gastrin-releasing peptide for transmission. When viewing these results together with our current findings, an interesting formation of an ET-1-responsive itch circuit emerges that has its origin with ET-1-responsive keratinocytes that use TRPV4 as critical Ca^2+^ influx mechanism in response to ET-1 receptor-A activation (10). Subsequently these ET-1-responsive keratinocytes activate innervating peripheral sensory neurons, which need to be more precisely defined in future studies, which in turn relay to spinal cord dorsal horn neurons, more than half specifically dedicated to itch-relay via neurotransmission that relies on gastrin-releasing peptide (16).

In another recent study from these same authors, *Trpv4* pan-null mice were reported to scratch less in response to intradermal injection with serotonin (56). The authors report that they did not see different scratch behavior in response to histamine. In this respect, their results differ from our results in *Trpv4* pan-null mice. This discrepancy may be related to technical detail such as difference in doses of pruritogen, animal ages, and behavioral assessment methods. Of note, the originating line of mice used is identical between our current and the referenced study. We believe that this seemingly perplexing discrepancy can possibly be resolved in future studies that focus on the influence of genetic background on nocifensive and pruritic behavior and, more importantly, on the impact that epigenetic regulation might play. Of note, different phenotypes of identical lines of *Trpv4* pan-null mice, propagated in different laboratories, have been reported previously (57, 58). Importantly, we want to stress that the focus of our present investigation is the distinct contribution of TRPV4 channels in keratinocytes to histaminergic itch, a subject of basic science and translational-medical relevance that is not directly approached in Akiyama *et al.* (56).

With TRPV4 expression in the primary sensory neuron and in the CNS in neurons and glial cells established, which roles do neural and neuronal TRPV4 play in itch transduction, transmission, and plasticity? Whereas these questions remain to be answered in future studies, we wish to reiterate the key concept of TRPV4 as forefront pruriceptor TRP channel functioning in epidermal keratinocytes, to drive the organismal scratch response. This concept bears the translational-medical mandate, as mentioned, to develop selective anti-TRPV4 treatments that can be applied topically and that will also have to be inert regarding epidermal cell growth regulation in view of recent findings of attenuated TRPV4 expression in skin epithelial malignancies (59).

Pruriceptor-TRPs comprise TRPA1, TRPV1, TRPV3 (40, 60–68), and now also TRPV4. Their possible mechanisms of interaction and the respective cellular locale will be attractive subjects for the following chapters in this intriguing story. Possibly, human genetic variation in the respective *TRP* pruriceptor genes might be relevant for different itch susceptibilities both for physiologic and pathologic forms of itch.

## Author Contributions

Y. C. and W. B. L. conceived and coordinated the study and analyzed the data. Y. C., Q. F., and Z. W. designed, performed, and analyzed the experiments. J. Y. Z. contributed reagents. Y. C., J. Y. Z., A. S. M., R. P. H., and W. B. L. drafted and revised the article. All authors reviewed the results and approved the final version of the manuscript.

## Footnotes

3 The abbreviations used are:

UVB: ultraviolet B
TRPV4: transient receptor potential (TRP) vanilloid 4
ET-1: endothelin-1
tam: tamoxifen.
